# Knowledge sharing in global health research – the impact, uptake and cost of open access to scholarly literature

**DOI:** 10.1186/s12961-017-0235-3

**Published:** 2017-08-29

**Authors:** Elise Smith, Stefanie Haustein, Philippe Mongeon, Fei Shu, Valéry Ridde, Vincent Larivière

**Affiliations:** 1 École de Bibliothéconomie et des Sciences de l’Information, Université de Montréal, Montréal, QC Canada; 2 Sciences Humaines Appliquées (Option Bioéthique), Médecine Sociale et Préventive, Montreal, QC Canada; 3 School of Information Studies, McGill University, Montréal, QC Canada; 4 École de Santé Publique, Département de Médecine Sociale et Préventive, Université de Montréal, Montréal, QC Canada; 5 Observatoire des Sciences et des Technologies (OST - CIRST), Université du Québec à Montréal, Montréal, QC Canada; 6 Université de Montréal Public Health Research Institute (Institut de Recherche en Santé Publique (IRSPUM)), Université de Montréal, Montréal, QC Canada

**Keywords:** Open access, Global health research, Knowledge translation, Publication ethics, Research impact, Research capacity, Publication policy

## Abstract

**Background:**

In 1982, the *Annals of Virology* published a paper showing how Liberia has a highly endemic potential of Ebola warning health authorities of the risk for potential outbreaks; this journal is only available by subscription. Limiting the accessibility of such knowledge may have reduced information propagation toward public health actors who were indeed surprised by and unprepared for the 2014 epidemic. Open access (OA) publication can allow for increased access to global health research (GHR). Our study aims to assess the use, cost and impact of OA diffusion in the context of GHR.

**Method:**

A total of 3366 research articles indexed under the Medical Heading Subject Heading “Global Health” published between 2010 and 2014 were retrieved using PubMed to (1) quantify the uptake of various types of OA, (2) estimate the article processing charges (APCs) of OA, and (3) analyse the relationship between different types of OA, their scholarly impact and gross national income per capita of citing countries.

**Results:**

Most GHR publications are not available directly on the journal’s website (69%). Further, 60.8% of researchers do not self-archive their work even when it is free and in keeping with journal policy. The total amount paid for APCs was estimated at US$1.7 million for 627 papers, with authors paying on average US$2732 per publication; 94% of APCs were paid to journals owned by the ten most prominent publication houses from high-income countries. Researchers from low- and middle-income countries are generally citing less expensive types of OA, while researchers in high-income countries are citing the most expensive OA.

**Conclusions:**

Although OA may help in building global research capacity in GHR, the majority of publications remain subscription only. It is logical and cost-efficient for institutions and researchers to promote OA by self-archiving publications of restricted access, as it not only allows research to be cited by a broader audience, it also augments citation rates. Although OA does not ensure full knowledge transfer from research to practice, limiting public access can negatively impact implementation and outcomes of health policy and reduce public understanding of health issues.

## Background

The 2014 Ebola outbreak proved disastrous for nations such as Guinea, Sierra Leone and Liberia, which were already rife with civil unrest [1]. Yet, in 1982, knowledge that Liberia had a high potential of endemic Ebola had been published in the *Annals of Virology* [2]. Local public health institutions and officials were most likely unaware of these findings as they remained hidden behind a paywall in a subscription only journal. This may have contributed to the lack of preventative measures which could have mitigated the severity and magnitude of the eventual outbreak [3]. During the outbreak, understanding the evolution of the epidemic from an epidemiological standpoint was, in itself, difficult given the lack of investment in data collection, sharing and management [4].

As mentioned by WHO in their report entitled Research for Universal Health Coverage [5], accessible knowledge is an important first step in the translation of knowledge from research to policymakers and stakeholders in low- and middle-income countries (LMICs). Although access to knowledge would not of itself have prevented or averted the Ebola epidemic, better informed health officials might have taken timely preventive measures and been better equipped to mitigate risks during and after the outbreak [6]. Actually, in the recent Zika virus outbreak, research is more readily accessible, providing evidence-based knowledge faster to mitigate immediate and future harms [7]. This may be due to consensus reached by important stakeholders (*British Medical Journal*, the *Nature* journals, the *New England Journal of Medicine*, and the seven *PLoS* journals) during the 2015 WHO consultation promoting sharing of data, results and pre-prints during public health emergencies [8].

As international healthcare research has evolved over the past several decades, so has the sharing of knowledge. Prior to the 1990s, the involvement of multiple international collaborators was much less prevalent and usually limited to matters of complex disease control (e.g. smallpox) [9]. Generally, the scope of ‘public health’ was determined by the resource capacity and geographical reach of a specific country or community [10]. It was common practice for researchers from high-income countries (HICs) to study health issues in LMICs. However, this type of research in the field of ‘international health’ was exclusive in that it rarely included or considered the interests and needs of researchers and communities in LMICs [11]. Knowledge remained with HIC research groups and was published in subscription journals, held behind ‘paywalls’ – expensive subscriptions or toll access, affordable mainly for HIC researchers and/or institutions. Restricting access to knowledge from public health research that can have direct influence in life or death contexts remains a serious social justice concern [12].

The contemporary approach to global health research (GHR) promotes partnerships that meaningfully include researchers and communities from LMICs [13]. Research ethics has developed benchmarks for ethical global research to minimise exploitation of local players by including them and giving them fair recognition in collaborative research partnerships [14]. Mutual capacity building is encouraged so that researchers from both HICs and LMICs may learn from each other [15, 16]. These partnerships can facilitate knowledge translation among the diverse actors in LMIC health research, including researchers, non-governmental organisations and healthcare providers [17]. The intended outcome is greater health equity on a global scale among people and nations [10]. Since researchers in GHR are called upon to work in a collaborative fashion for health equity, sharing knowledge on a global scale is of central importance [6, 16, 18].

There are different ways to increase access to published knowledge. In LMICs, there are programmes such as the Health Internetwork Access to Research Initiative (HINARI), an initiative put together by WHO in collaboration with journal publishers, which provides greater access to many different research resources including e-books, textbooks and up to 14,000 journals, many of which are subscription based [19]. HINARI’s goal is to contribute to improving world health [19]. HINARI promotes ideals central to GHR, such as furthering equity in research access, but it does have important practical limitations. While HINARI provides free access to research institutions in low-income countries, it still requires that medium-income countries pay ‘low cost fees’ (US$1500 per year) for full access to HINARI resources [20]. Although these fees are indeed lower than the full price for HINARI resources through subscriptions or toll access, certain institutions – notably those that play many other roles such as healthcare provision and health prevention and promotion – have competing claims for limited funding, may not prioritise research funding within their institution, or may simply not have the necessary funding.

Moreover, since HINARI is a voluntary programme, publishers may choose to opt out or restrict free access status to specific countries; this creates uneven and uncertain access for users. This instability was well exemplified in 2011, when five publishers withdrew free access to more than 2500 biomedical and health journals including Elsevier’s *Lancet* journals from Bangladesh [21]. Although free access was soon reinstated after public outcry, the sustainability of such initiatives led by for-profit publishers remains questionable [22]. In fact, a similar withdrawal of access to various publishers in Nigeria in 2013 and 2014 has had the effect of reducing HINARI users in Nigeria [23]. It is noteworthy that the subscription journals are published almost exclusively by Western publishers such as Wiley, Taylor & Francis, Springer Nature, and Elsevier; the consequence of this is a financially induced knowledge inequality focused in the places where the research could have the highest impact.

Open access (OA) is another method where scholarly content is freely available online to all readers. Research has shown that OA is associated with higher citation rates [12, 24–26], likely as a consequence of wider accessibility. One main issue of certain high-profile OA journals is the existence of significant article processing fees (APCs) paid by authors, which may disadvantage researchers who are unable to cover these costs, emphasising the already significant inequity in research dissemination [27]. Although many funding institutions or universities may cover APCs [28], this is not systematically the case, especially in LMICs. To offset this financial barrier, certain journals offer OA waivers for researchers in low-income countries and in certain middle-income countries [29]; however, criteria to obtain waivers differ based on the journal.

It must be noted at the outset that the OA model has also led to the creation of a number of ‘deceptive publication practices’ often referred to as ‘predatory journals’ that do not follow standard peer-review process and often lack quality and transparency [12]. To ensure a level of quality control, journals do traditionally have an important role in managing the peer-review system where experts critically review research before it is made public. Regardless of the journal model – whether subscription based or OA – a certain level of peer-review is seen as essential [30].

Free access to knowledge may also be provided when researchers self-archive their papers, as we often see in public or institutional repositories. The copyright transfer agreements of many journals allow for the archiving of pre-prints and/or post-prints of journal articles, a practice termed ‘green OA’. Journal policies that do not allow for self-archiving often have an embargo period during which they control access to peer-reviewed articles for a specific range of time (generally 6 months to 1 year in journals publishing GHR papers); the impetus behind such embargos is to require institutions to purchase and thus fund subscription-based journals.

Over the last few decades, many studies have analysed the evolution of the OA availability of papers [31–36]. When one combines all different forms of OA, 50% of all biomedical research papers published between 2004 and 2011 were freely available in 2013 [24]. The same study shows that for the field of Public Health and Health Services, of which GHR can be considered a subfield, the share of OA is slightly higher, with 57.2% [24]. Given the importance of worldwide knowledge access in GHR, one might expect OA to be more prevalent in this field than in others. However, some may consider APCs to be simply too costly. The goal of this article is to (1) quantify the uptake of various types of OA used in GHR research, (2) calculate the financial costs of such practices from the authors’ point of view (paying for APCs), and (3) assess the impact of different OA models as indicated through citation analysis. Although there exist many other elements that influence the use of OA in GHR, such as journal prestige, general awareness, funder requirements and availability of repositories, these aspects are outside the scope of this specific research. Since the main goal of this paper is to assess the differences between different types of OA models, the comparison of subscription costs paid by university libraries and OA costs known as APCs paid by the author(s) is beyond the scope of this paper.

## Methods

The PubMed search engine was used to retrieve all research articles indexed under the Medical Subject Heading (MeSH) ‘Global Health’ from 2010 to 2014 [37]. From 1978 to 2014, research in global health was indexed under ‘International Health’; however, with the increase of institutions, research and journals in GHR [38], ‘International Health’ was replaced by ‘Global Health’ in 2015. This modification can be explained by the historical shift described at the outset of this paper, in which researchers wished to create global partnerships based on global equity. The analysis is based on 3366 GHR journal articles published in 909 journals. OA availability was defined at journal level (Table 1) as well as paper level (Table 2).

**Table 1:** Definition of access categories at the journal level

Category	Definition	Article processing fees	Subscription fees	Embargo
Gold OA journal (no APC)	OA journal that provides immediate access to all of their content free of charge to both readers and authors	No	No	No
Gold OA journal (with APC)	OA journal that provides free immediate access to all of their content based on an author-pays model via APCs	Yes	No	No
Delayed OA journal	Subscription journal that provides all content for free after an embargo period, which differs depending on the journals; journals that provide delayed OA to only some of their content were classified as subscription journals and their free papers identified as delayed OA articles	No	Yes	Yes
Hybrid journal	Subscription journal that is primarily financed by the reader-pays model based on subscriptions and pay-per-view fees, but allows authors to pay an APC to make their article available free of charge for the reader without delay	Sometimes	Yes	Sometimes
Subscription only journal	Subscription journal that is financed by reader-pays model based on subscriptions and pay-per-view fees and does not offer author-pays OA options	No	Yes	Yes
Unknown	Journal for which the access status could not be determined	Unknown	Unknown	Unknown

*APC* article processing charge, *OA* open access

**Table 2:** Definition of access categories at the paper level

Category	Definition	Article processing fees	Subscription fees	Embargo
Gold OA article (no APC)	Article published in gold OA journal website when published; instantly available on journal website when published	No	No	No
Gold OA article (with APC)	Article published in gold OA journal; instantly available on journal website when published	Yes	No	No
Delayed OA article	Free article published in delayed OA or subscription journal with delayed OA option	No	Yes	Yes
Hybrid article	Free article published in a subscription journal	Yes	Yes	No
Green OA article	Articles in subscription or hybrid journals which have been self-archived by the author or affiliated institution to provide free access	No	No	No
Other free access	Free article in subscription journal or journal for which the status is unknown	Unknown	Sometimes	Sometimes
Toll access	Article that can only be accessed through a subscription or pay-per-view model and has not been self-archived	No	Yes	Yes
Paper status unknown	Article for which the access status could not be determined	Unknown	Unknown	Unknown

*APC* article processing charge, *OA* open access

The OA status of a journal was determined using the Directory of Open Access Journals, Ulrich’s Periodicals Directory and journal lists from Elsevier, Sage, Springer Nature, Taylor & Francis, and Wiley-Blackwell. Due to conflicting and missing information, the status of each journal was verified and APCs retrieved from the journal websites. APCs were collected in or converted to USD. If APCs were not provided in USD, currencies were converted using the mean of weekly historical conversion rates between 1 January 2010 and 31 December 2014 using OANDA [39]. To understand who benefits financially from OA, we identified publishers of journals (as seen in [40]). The SHERPA RoMEO database was used to determine whether self-archiving was formally supported or not. Self-archiving may come in different forms, including pre-print (i.e. before peer-review), post-print (i.e. after peer-review) or in the final PDF formatted by the journal publishers. We did not verify which type of manuscript versions were shared (e.g. pre-print, post-print), we only distinguished between journals that did or did not allow self-archiving.

At the paper level, OA availability was determined using the journal level information together with PubMed search as well as a manual Google search for self-archived versions of articles published in subscription journals. Since the main criteria is ‘availability’ of research, we included articles online from various platforms such as institutional repositories (e.g. university, research centre), publicly based repository (e.g. PubMed Central, Europe PMC, SciElo, BIREME), venture-capital based online social networking sites that allows self-archiving (e.g. academia.edu, researchgate.net), and private pages owned by researchers. Since many closed access journals allow for self-archiving, we compared which toll access papers could be self-archived but were not.

A citation analysis was carried out at the paper level to compare the scholarly impact of different types of access categories. Citations were obtained from the Web of Science (WoS), thus restricting the set of papers from 3366 to 2655 papers. These citations were then normalised by year. A normalised citation rate of 1.0 thus indicates that a paper (or a set of papers) was cited according to the expected average citation rate for the set of GHR papers published in a particular year. A citation rate above 1 indicates impact above average and a citation rate below 1 indicates impact below average.

Using institutional affiliations of authors appearing on citing articles, an analysis was conducted to explore whether certain accessibility categories are cited more or less predominantly by various countries depending on their socio-economic context. Countries were categorised into (1) low-, (2) lower middle-, (3) upper middle- or (4) high-income countries [41]. A citing paper written by authors from different countries was assigned once per World Bank Atlas (WBA) country group. Countries were retrieved manually for 374 citing papers, for which the WoS did not include any address information. Two citing papers were excluded because the authors’ addresses could not be determined and 50 citations from Guadeloupe, Netherlands-Antilles, Palestine and Reunion were excluded because they were not classified by the WBA. The analysis of citing countries was normalised in a way that each paper was weighted equally (as a percentage of citing papers) regardless of their actual number of citations, as we aimed to evaluate how the distribution of countries changed per category. In other words, the over- and under-representation of a WBA group among citations per access category was calculated as the average percentage per paper per access category divided by the overall average percentage of that WBA group. Research was conducted using publicly available data and, as such, is not considered ‘research on human subjects’; such research is exempted from institutional review board ethics approval. Datasets used for this study are available online [42].

## Results and Discussion

### Uptake of OA

**Fig. 1:**
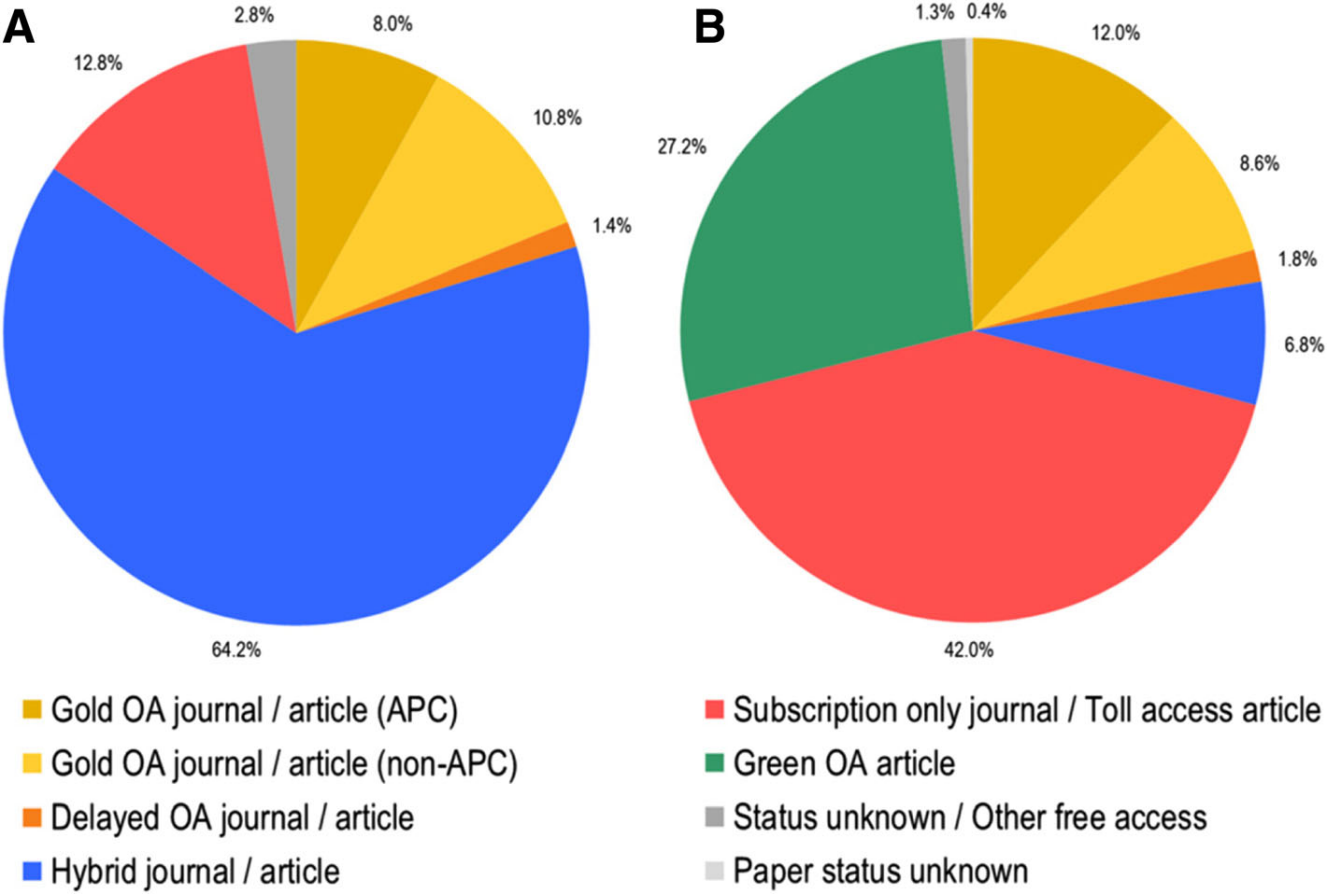
Percentage of journals (**a**) and articles (**b**) per type of access category

From 2010 to 2014, 909 journals published at least one paper indexed as GHR. Figure 1 provides an overview of the frequency of different access categories at the journal (Fig. 1a) and paper level (Fig. 1b). Including both those with and without APCs, 18.8% journals and 18.6% papers are available through gold OA. While there are less gold OA journals with an APC, the percentage of papers published in these periodicals (12.0%) exceeds those in gold OA journals without an APC (8.6%), most likely because they are more renowned and have impact factors (IFs) (such as *Lancet Global Health*, *PLOS Medicine* and *Global Health Action*). The majority of journals (64.2%) are hybrid, meaning that authors can choose to publish research in closed access without financial cost or provide an APC and make the paper OA; still, only 6.8% of papers make use of the hybrid OA option. If immediate OA access is sought via the publisher, GHR researchers seem to choose gold journals over hybrid ones. On the paper level, 69.2% of all GHR publications are not available for free on the publishers’ website. However, 27.2% of toll accessed papers are self-archived (also called green OA) leaving a total of 42.0% of GHR papers only accessible through subscription or toll access.

According to the archiving policy recorded by RoMEO, 84.0% of the 700 subscription and hybrid journals allow green OA, while 7.4% explicitly prohibit it (8.9% of the 700 subscription and hybrid journals were not graded by RoMEO). Among RoMEO-graded subscription and hybrid journals, we determined that 733 papers were self-archived in accordance to journal policy and 1139 were not self-archived, despite authors having the possibility of doing so according to the journal policy. This shows that 60.8% of papers that could have been self-archived were not. In a field where OA seems of practical and ethical importance for the sharing of knowledge promoting health equity, it is surprising that researchers do not make their papers available when they are legally able to do so without any cost; this suggest that authors might not be aware of green OA policies.

### Cost of OA

**Fig. 2:**
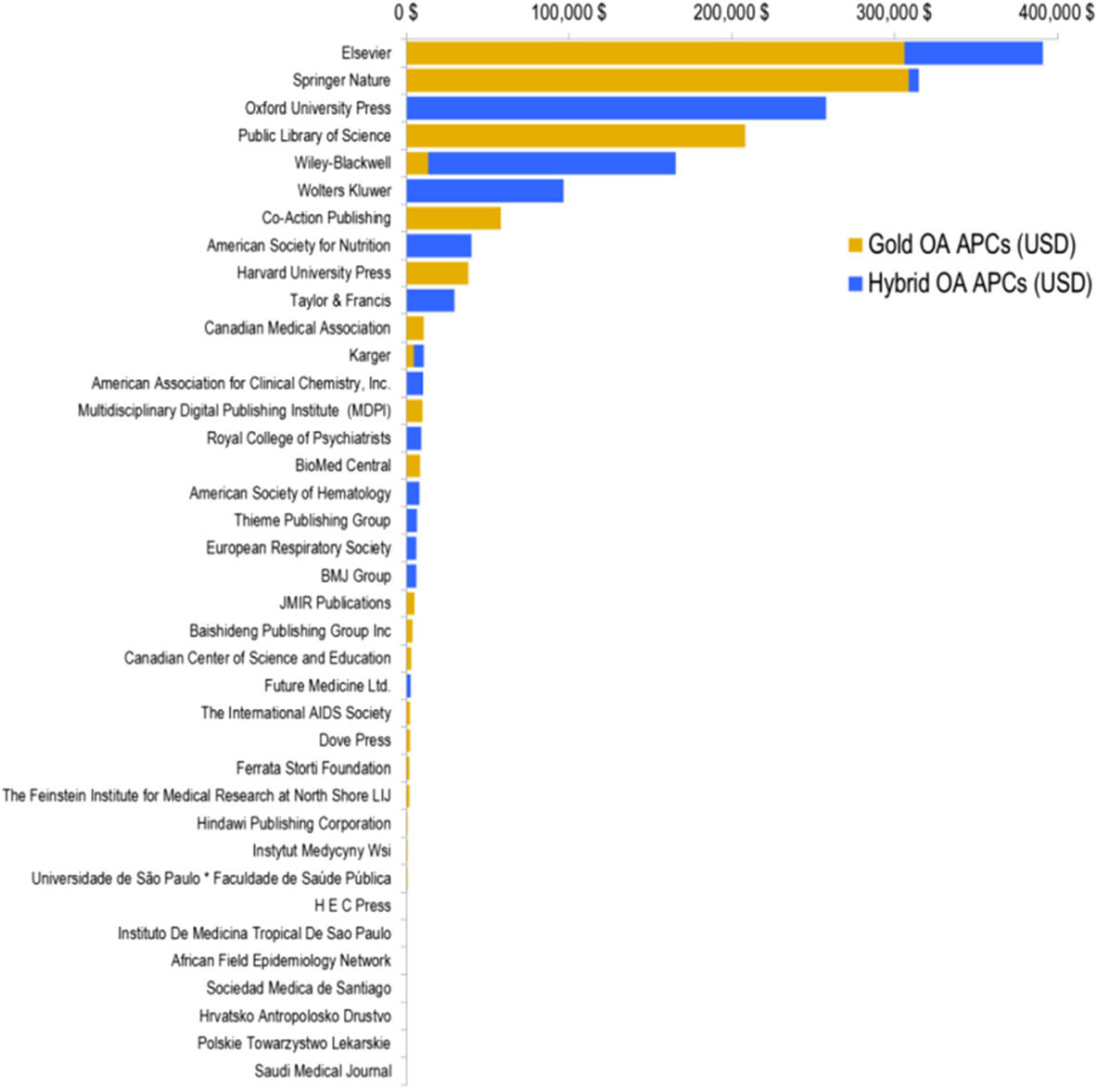
Sum of gold and hybrid article processing charges per publisher

Figure 2 presents the total APCs required for GHR publications over the 2010–2014 period, by publisher and OA category. The total fees amounted to US$1.7 million for 627 gold OA (APC) and hybrid papers; on average, authors paid US$2732 (SD = US$1090) to make their publication freely available on the publisher’s website. These APCs can be explained by many factors, such as the high scholarly capital associated with publishing in journals of big publishers (which are generally hybrid), as well as the presence of an oligopoly in the academic publishing system [40]. Such oligopolistic conditions create a limited market, reducing economic competition between publication houses and giving little incentive to decrease prices. More specifically, according to our findings, 93.4% of APCs were paid to journals owned by the 10 most prominent publishing companies. Elsevier alone accounts for 22.8% of the total APCs and charged the highest average gold APCs (on average US$4435 for 69 papers) among all publishers in the GHR set. Their APCs for 26 hybrid fees were lower and close to the GHR average at US$3271 per paper; nevertheless, Elsevier’s hybrid uptake remained low at 3.5%.

Mean OA fees in hybrid journals (US$3240 per paper) are higher than those in gold OA journals (US$2452), which is somewhat surprising given that the former group of journals already has revenues from subscriptions [43], while APCs are the main source of revenues of the latter. Historically, hybrid journals have justified this double income stream as a way to reduce subscription fees proportionally with the uptake of OA [44]. However, this fee reduction has been questioned given the lack of transparency of journal costs and the growing fees of both APCs and subscriptions [45]. This lack of transparency augments the possibility of a phenomenon of ‘double dipping’, in which journals profit from both revenue streams – APCs and subscriptions – without readjusting the price based on APC uptake [46]. Even though APCs are getting considerably expensive, they continue to be promoted by many important stakeholders and funders making gold and hybrid OA publishing a growing business [47].

### Impact of OA

**Fig. 3:**
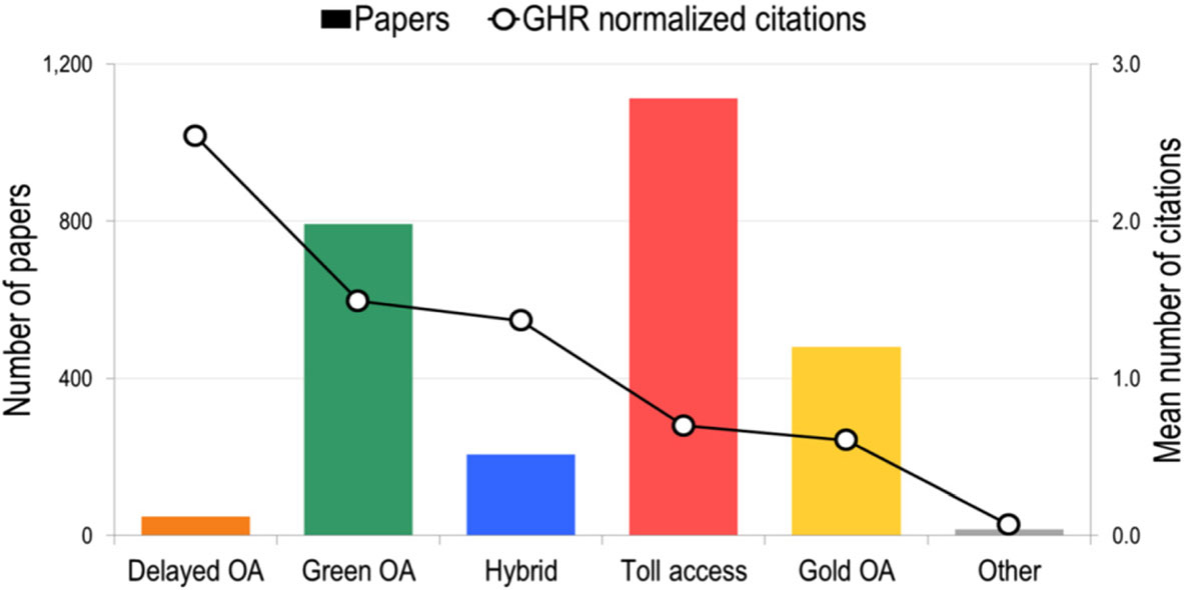
Citation impact and number of papers per access category

Figure 3 demonstrates the number of papers and the mean number of citations per type of OA. Articles categorised as delayed, green and hybrid OA are cited above average while toll access and gold OA papers are cited below average. Of particular interest is the difference between green OA (1.5) and toll access (0.7), which shows that self-archived papers receive more than twice as many citations as those hidden behind a paywall, which corroborates previous findings obtained in other fields [48, 49]. It should be noted that the green OA articles available on PubMed Central were cited more (1.9) than those deposited on other platforms (1.3).

Hybrid articles were cited 37% more than the average GHR paper and twice as much as toll access articles, which supports the previous findings that OA broadens citation impact. However, one has to keep in mind the paramount fees for hybrid publishing, while self-archiving comes at no charge to the author and a higher increase in impact. Although proper IT infrastructure and human resources are necessary to ensure an organised, indexed and sustainable repository, studies show that such costs are meager compared to subscription or gold and hybrid OA (e.g. [35, 50]). Articles published in gold OA journals remained cited 40% below the average GHR paper, with no difference between APC and non-APC journals. The fact that the impact of gold OA papers is lower than those published in subscription journals (green, hybrid, toll) can be partly explained by the fact that prestigious journals are largely subscription journals, while many gold OA journals are younger and, thus, are not as prestigious. Journal prestige is an important confounding factor that limits this type of study [51]. Results for delayed OA and other papers are based on as few as 49 and 16 papers, respectively. Given this limited number, results are inconclusive.

**Fig. 4:**
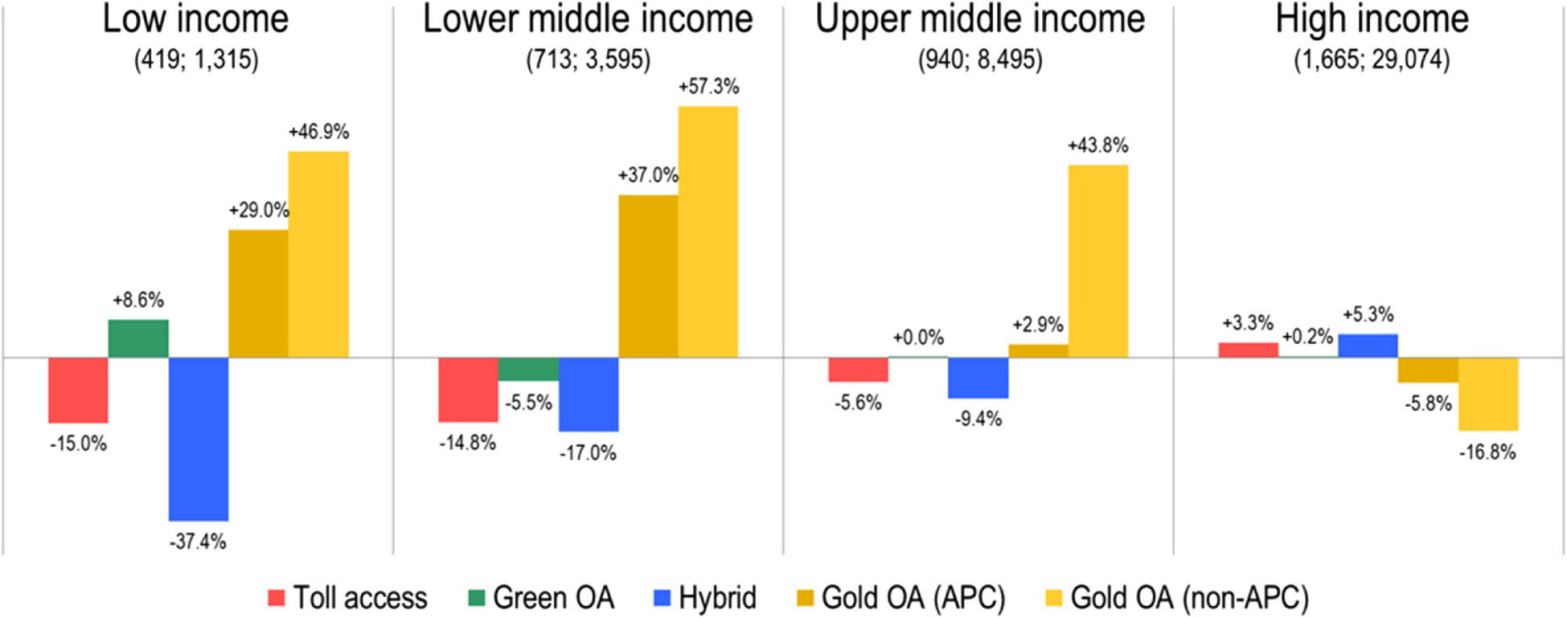
Over- and underrepresentation of citing countries by World Bank Atlas (WBA) country classification (a, low-income countries; b, lower middle-income; c, upper middle-income; d, high-income countries) and access category. Numbers in parentheses represent the number of papers cited by each country category as well as the number of citing WBA category-cited paper combination

Usage of GHR papers varied according to the socioeconomic situation of countries (Fig. 4). Indeed, 3.1% of the 42,479 citing WBA category-cited paper combinations came from low-income countries, 8.5% from lower middle-income, 20.0% from upper middle-income and 68.4% from HICs. Such underrepresentation of researchers from LMICs is well-known [52, 53]. Analysing the average share of citing countries per paper, researchers from low-income countries were, on average, 29.0% and 46.9% more likely to cite papers from gold OA journals with and without an APC and 8.6% more likely to cite a green OA paper, while they were underrepresented on papers citing hybrid (–37.4%) and toll access papers (–15.0%). The underrepresentation of LMICs on papers citing hybrid papers show that, even with availability on the publisher’s website, such articles are rarely considered by LMIC researchers. This may simply be the result of subscription journals not traditionally being accessible and thus researchers are not in the habit of searching in such resources. The results for HICs suggest that the type of access has less influence on HIC authors. However, they are underrepresented on papers citing articles published in gold OA journals with (–16.8) and without (–5.8) APCs, which might again be explained by the lower prestige of these journals in comparison to many traditional subscription-based journals.

### Limitations

The first limitation of this study pertains to the calculation of green OA articles at the paper level. After we compiled the number of PubMed articles that were freely available in green OA format, we performed a manual search on Google to assess other articles that may be freely accessible. We used the author’s name and the title of the article, and then looked at the top ten results to find a green OA article. We found many articles on institutional repositories, publicly based repositories and social media sites. However, there may be some available research that was simply not found by our search method.

The second limitation is regarding the calculation of costs (APCs) paid by authors. In cost calculations, we did not include waivers or institutional discounts. Waivers can be given to cover part or all of APCs where funding is limited, especially in the case where a researcher is affiliated to an institution in a LMIC. Waivers are generally granted on a case by case basis and are not made public. Institutional discounts were also not included in our calculation because they are quite variable based on institution and year. In our study, APC costs were gathered in 2016, but APCs were paid before that time and thus may have been different – most likely slightly lower due to inflation. Given all these factors, the total APCs may be slightly overestimated herein.

The third limitation pertains to the categorisation of citing papers written by authors from different countries once per WBA country group. This decision was made because it is not possible to know how much contribution each author made to the paper. We could (1) fractionalise by number of authors or country group according to WBA classification or (2) count once for each author or country group. Since we analyse on the country level (or country group according to WBA) we do not wish to risk punishing papers with multiple authors that are in more than one country groups.

The fourth limitation is that we decided to compare average citation rate of different access levels without controlling for IF. We decided not to include the IF as it is a flawed indicator already overused in the scientific community [54]. It is also discipline-specific and not readily comparable between fields. Additionally, in this specific study, the majority of the 909 journals published only one or a few papers; such an analysis would be performed on very small amounts of data. However, herein, we did make the logical assumption that closed journals will most likely have more prestige because prestige is built with time, something that is not yet acquired with novel OA journals. Yet, there is no empirical evidence-based research to validate this last assumption.

## Conclusions

This paper explored publication practices of GHR researchers, a field where sharing of knowledge is inherent to its mission of equity in healthcare and essential to its collaborative nature. Regardless of this emphasis on sharing, our research shows that 42.0% of scholarly articles are not freely available online even if many funders, scholars and universities promote some form of OA (mainly green or gold). While it is understandable that researchers gravitate towards traditional, highly reputable journals, it remains sobering to note that only 39.2% of papers published in journals that allow green OA, which comes at no cost for the authors, were in fact self-archived. Findings clearly show that self-archiving does not only promote knowledge sharing but also increases the impact of research. Many reasons could explain this behaviour, such as a lack of knowledge of journals’ self-archiving policies, lack of appropriate user-friendly self-archiving platforms, lack of time or general unawareness of the advantages of green OA (i.e. such as increased impact). Researchers may think that publication in traditional closed (paywalled) journals are sufficient because of initiatives such as HINARI, which provide a certain level of free or low cost access to research for LMIC researchers.

Despite increased access provided by HINARI, LMIC researchers are still underrepresented in citing subscription journals. Our study supports the claim that increased access through green and gold OA is reaching underrepresented researchers more so than subscription journal articles. As such, it provides more research capability that is at the centre of GHR. When researchers are to publish their work in an accessible format it is important to choose an OA type that best suits their needs and not assume that the most expensive APC has the best impact, reach and citability. In fact, hybrid OA journals, which have the most expensive APCs, were the most underrepresented in LMICs. It remains unclear why APCs for hybrid journals remain higher than gold APCs given the fact that these journals also ask for subscription fees.

Since the APCs are mainly paid to the ten same publishers creating an oligopoly, there is little incentive to keep APCs low. This oligopoly may also run much deeper than costs; it creates an important inequity in publication. Although publishers may wish to include researchers from LMICs through waivers, they have not really included LMICs in the publication industry itself. After witnessing significant inequities and issues related to exploitation in global health, the impetus behind GHR was to provide a space for equal partnerships. Broadening these partnerships to the publication industry, which is a significant gatekeeper in research, may provide for a stronger voice for researchers in LMICs with the goal of reducing power inequities in global health more broadly.

## Abbreviations

APC: article processing charge
GHR: global health research
HIC: high-income countries
IF: impact factor
LMIC: low- and middle-income countries
OA: open access
WBA: World Bank Atlas
WoS: Web of Science
